# 

**Published:** 1883-01

**Authors:** 


### *Dental Department of the University of California.*

The Dental Department of the University of California held its *first* commencement exercises at B'nai B'rith Hall, San Francisco, on Wednesday evening, November 8th, 1882.

An address on behalf of the State was delivered by Governor Geo. C. Perkins, and also one on behalf of the Board of Regents by Rev. Horatio Stebbins, A. M., D. D.

The valedictory on behalf of the Faculty was delivered by the Dean, Prof. S. W. Dennis, M. D., D. D. S., and that on behalf of the graduating class by Henry John Plomteau.

The number of matriculates was thirty-two.

The degree of D. D. S. was conferred on the following members of the graduating class by W. T. Reid, A. M. President of the University.

Thomas Watson Hall, Oakland, Cal.; Charles Wesley Hibbard, San Francisco, Cal.; Thomas Morffew, San Francisco, Cal.; Henry John Plomteaun, Oakland, Cal.; Charles Wesley Richards, San Fracisco, Cal.; Wm. Henry Stanley, San Francisco, Cal.; Gustav William Sickel, M. D. San Francisco, Cal.; August Van Crombrugghe, San Francisco, Cal.;

Unlike the Eastern colleges the Medical and Dental Departments of the University of California hold their sessions in summer instead of in winter.

### To Readers and Correspondents.

Communications intended for publication, and exchange journals and papers, should be addressed to the EDITOR OF THE AMERICAN JOURNAL OF DENTAL SCIENCE, 259 N. Eutaw St., Baltimore, Md. All letters relating to the business of the journal or containing cash remittances, to be directed to Messrs. SNOWDEN & COWMAN. Articles for publication should be received by the editor at least three weeks previous to the first of the month on which the number in which it is designed for them to appear, is issued.

### BIBLIOGRAPHICAL NOTICES.

Tafts Practical Treatties on Operative Dentistry..........................427
Elements of Dental Materia Medica and Therapeutices..........................428
The Pharmacopœia of the United States of America..........................428

### MONTHLY SUMMARY.

Resolution of Alveolar Abscess into Serous Cyst..........................429
A New Remedy for Spasms..........................430
Hemorrhage after Teeth Extraction..........................431
Treatment of Diptheria..........................432

---

☞ The American Journal of Dental Science will contain fifty pages of reading matter in each number, making a volume of about

*Six Hundred* pages,

EXCLUSIVE OF ADVERTISEMENTS.

THE

### AMERICAN JOURNAL

OF

### DENTAL SCIENCE.

The *Journal* is issued on the first of each month and contains not less than *fifty* pages of reading matter in each number, exclusive of advertisements, making a volume of *six hundred* pages per annum.

In each number will be found Original Communications, Correspondence, Selected Articles, Monthly Summary, Bibliographical Notices and Editorial Matter, and every effort will be exerted to render the *Journal* worthy of the patronage of the Dental profession.

A generous co-operation is respectfully solicited from the members of the profession; and as the *Journal* has now a printing office of its own, which materially lessens the cost of its publication, it will be issued at the reduced subscription price of

**$2.50 Per Annum in Advance.**

Communications are respectfully solicited on all subjects pertaining to the practice of dentistry, as well as reports of cases occurring in dental practice.

### RATES OF ADVERTISING.

| | 1 MO. | 2 MO. | 3 MO. | 6 MO. | 12 MO. |
|---|---|---|---|---|---|
| 1 PAGE. | $15 00 | $22 50 | $30 00 | $50 00 | $100 00 |
| ½ " | 10 00 | 15 00 | 20 00 | 30 00 | 50 00 |
| ¼ " | 7 00 | 11 00 | 15 00 | 20 00 | 30 00 |
| ⅛ " | 5 00 | 8 00 | 11 00 | 15 00 | 20 00 |

All original communications designed for publication, works for review, new instruments for notice, exchanges, &c., should be directed to the editor, 259 N. Eutaw St., Baltimore, Md.

All advertisements and subscriptions should be addressed to

SNOWDEN & COWMAN,

*Publishers of the Am. Journal of Dental Science.*

86 W. FAYETTE ST. BET. CHARLES & LIBERTY STS.,

BALTIMORE, MD

